# Retrospective and Prospective

**Published:** 1888-12

**Authors:** W. C. Barrett

**Affiliations:** No. 208 Franklin St., Buffalo


### RETROSPECTIVE AND PROSPECTIVE.

With this number the INDEPENDENT PRACTITIONER ceases to exist. Not that the good old journal is to die—quite the reverse. But with the January number it will assume a new name, and be known no longer by the old title. When the chrysalis bursts its cocoon and emerges the winged butterfly, it is perhaps but proper that it should henceforth bear a title which will not recall its former life. And yet as one who loved the old journal and its old name, who labored for years to help in spinning that cocoon, who gave to the work all his hours of recreation and many of those properly belonging to needed rest, who found his pleasure and reward in the work itself, and the consciousness that he was assisting to build up the profession to which his life was devoted, the former editor cannot witness the change without some feelings of regret. From a very small beginning he had seen the journal grow with its added years, and increase in strength and influence as it gained experience and knowledge, until in the estimation of the most intelligent dentists it stood second to none in rank or influence. The writer will have this consolation—that the INDEPENDENT PRACTITIONER will be permanently associated with his name, and it is a connection of which he will ever be proud. However great the INTERNATIONAL DENTAL JOURNAL shall become, and much as its fame may overshadow that of its progenitor, it will not entirely obliterate the recollections of the past, and herein is some comfort to be found, even though it be from a selfish view.

When the former editor looks back upon the history of this journal he is filled with astonishment. That men without journalistic experience should be able to steer clear of the thousand rocks in mid-channel which have proved disastrous to much better navigators is a piece of good luck which they had no right to anticipate. The only explanation of the measure of success which the journal attained under their management is found in the fact that the time was ripe for such an enterprise, and dentists ready to sustain such a work, even though it was pushed forward with more of zeal than ability. It was a transition period in our educational affairs. If one will look around and see what a great change in this direction has been wrought during the last few years—changes which the journal has labored with others to bring about—he will better comprehend that the period of its foundation as a dental journal was one which demanded the expressions to which the INDEPENDENT PRACTITIONER always endeavored to give voice. It was "independent" not only in name, but in fact; and though often mistaken, perhaps, it was generally believed that its errors were those of judgment and not of intention. The men who were connected with it were not believed to be seeking any ulterior objects of their own, but to be working for that which they believed to be the best good of dentistry. In this faith the journal was enabled to gather a corps of contributors and supporters such as any journal might be proud of. The best intelligence of dentistry was glad to write for its pages, and its readers comprised the very best men in the profession. It did not pretend to monopolize this intelligence by any means, for its worthy contemporaries could all, in greater or less degree, claim the same; but it was certain that none had any cause to boast over the INDEPENDENT PRACTITIONER.

The time came when the very success for which the members of New York Dental Journal Association labored so strenously served to defeat them. The labor became so great, the business interests so complicated and the responsibility so overwhelming, that they were unable to stagger under it longer, and it became necessary to find broader shoulders, and those which carried less of other burthens, to sustain this constantly increasing weight. In this emergency "The International Dental Journal Company" was formed, a man of commanding ability secured who could give to the business his undivided attention, and the old publishers, still retaining an interest in the journal, turned it over to a more vigorous and capable management. Six months of their work is now before the subscribers, and each is capable of forming an intelligent opinion concerning what the future will probably bring forth. There can be but one opinion; and that is that a wide field of usefulness opens before the Journal, and that the hands which now manage the helm are entirely competent to steer the craft amid even the wildest storms. The subscribers must see that the present editor has much greater facilities for serving them than had the former one, and that he is capable of giving them a journal which is much more worthy their patronage than it has been in the past. It is therefore the duty of every one to stand by him, and give him their active aid. Whether or not dentistry is to have a literature worthy of it depend upon them. The editor has disengaged himself from all other business, that he may give his whole attention to journalism; and it will be but a poor encouragement for such labors if dentists do not give him a fitting support. Every dentist who has the good of his profession at heart will therefore endeavor to sustain him in this, the first instance of the entire devotion of a competent man to dental journalism.

That the Journal under his management has doubtless now entered upon a career of honor and usefulness to which its past was but an introduction, no one will dispute. The undersigned has accepted a position as associate editor, although he prefers that his name should not appear upon the cover page, as the managing editor desires; but he will be only too glad when he can see an opportunity to serve the INTERNATIONAL DENTAL JOURNAL and its readers by contributing to its pages. W. C. BARRETT.

No. 208 Franklin St., Buffalo, Nov. 20th, 1888.

